# ACTH-Producing Neuroendocrine Carcinoma of the Liver with Cushing's Syndrome

**DOI:** 10.1155/2023/9946271

**Published:** 2023-09-26

**Authors:** Mudassar Sandozi, Saagar Pamulapati, Aniqa Zaidi, Zuzanna Stuart, Sneha Pamulapati, Ajay Doniparthi

**Affiliations:** ^1^Javon Bea Hospital-Rockton, Rockford, IL, USA; ^2^Midwestern University, Downers Grove, IL, USA; ^3^University of South Florida, Tampa, IL, USA

## Abstract

Paraneoplastic Cushing's syndrome arises when neuroendocrine tumors cause excess glucocorticoid production. We report a case of ectopic ACTH-producing liver neuroendocrine tumor. A 71 y.o. female with a history of rectal squamous carcinoma presented with fatigue and diffuse swelling. Liver biopsy revealed metastatic neuroendocrine carcinoma. Workup revealed markedly elevated morning cortisol and ACTH. Overnight dexamethasone suppression testing and positive immunostaining for ACTH on biopsy suggested paraneoplastic Cushing's syndrome secondary to neuroendocrine hepatic tumors with bony metastasis. This explained the patient's persistent anasarca, hyperglycemia, and electrolyte abnormalities. Despite multiple interventions, the patient's clinical status declined, and she expired.

## 1. Introduction

Neuroendocrine neoplasms are quite rare, constituting only about 2% of all malignancies. They can arise in most organs but are predominantly seen in the lung and gastrointestinal system [1]. In the GI system, commonly afflicted sites include the small intestine, pancreas, and appendix [2]. As these tumors originate from neuroendocrine cells, they have the potential to secrete various hormones, such as gastrin, serotonin, adrenocorticotropic hormone (ACTH), or somatostatin. Excessive hormone secretion can manifest as clinical syndromes. Paraneoplastic Cushing's syndrome, for example, results from excess glucocorticoid production. Cushing's syndrome has been well established in neuroendocrine tumors involving the lung but appears in sparse reports of ectopic ACTH production involving gastrointestinal-associated tumors [3]. Herein, we report an exceedingly rare case of ectopic ACTH-producing neuroendocrine tumor of the liver causing Cushing's syndrome.

## 2. Case Report

A 71 y.o. female with a past medical history of squamous carcinoma of the rectum, diastolic heart failure, type 2 diabetes mellitus, essential hypertension, hyperlipidemia, stage 2 chronic kidney disease, and peptic ulcer disease presented to the hospital with 4 days of progressive fatigue and diffuse swelling. During a previous hospitalization, CT abdomen/pelvis and follow-up MRI found an 11 mm hypoattenuating nodule near the hepatic dome, a 12 mm nodule in periphery of the right hepatic lobe, inferior to the dome, and an ill-defined 11 mm region of nodularity at the junction of segments 2 and 3 of the left hepatic lobe, all concerning for metastatic disease. Lateral limb of the left adrenal gland showed 3.1 × 1.6 cm nodular thickening with a cyst-like 12 mm low-attenuation lesion at the lower pole. Anemia was explained by peptic ulcer disease secondary to NSAID use for headaches, and she was discharged home with instructions to minimize her NSAID use and take pantoprazole 40 mg twice daily for 8 weeks. Soon after, her weakness progressed to requiring assistance from her husband for standing, and she was brought back to the hospital. Upon presentation to the hospital, she was unable to stand without assistance and had 4+ pitting edema in all four extremities. She was found to be hyperglycemic, hypokalemic, and hypocalcemic. Comprehensive metabolic panel revealed mild elevation of liver enzymes and evidence of protein malnutrition with AST-ALT of 34 : 74 U/L, alkaline phosphatase of 147 U/L, and albumin of 3.3 g/dL. Respective electrolytes were replaced and she was given nutritional supplementation, but her weakness persisted. She was discharged to an acute rehab facility but was readmitted after a sudden drop of hemoglobin to 5.8 g/dL and platelets to 109,000 per microliter. Hemoglobin was corrected to 11.0 g/dL after a 3-unit packed red blood cell infusion, although platelets remained low at 66,000 per microliter. Creatinine and BUN remained at baseline of 0.8 and 24 mg/dL, INR was 1.3, and PT was 12.6 seconds at this time.

Gastroenterology service was consulted for revaluation of anemia, increasingly elevated transaminases (AST-ALT 89 : 160 U/L), and acute thrombocytopenia. Nephrology was consulted for persistent electrolyte abnormalities and presence of anasarca. Metolazone 10 mg PO daily was added to Lasix; orders were placed for water restriction, continued electrolyte replacement as needed, and follow-up echo which showed a similar ejection fraction of 55-60% with grade 1 diastolic dysfunction. The IR team proceeded with a liver biopsy, which resulted in findings consistent with metastatic neuroendocrine carcinoma. Morning cortisol was elevated above 10 times the upper limit of normal at 196 mcg/dL, and ACTH was found to be more than 14 times the upper limit of normal at 920 pg/mL. Overnight dexamethasone suppression test revealed serum cortisol greater than 1.8 mcg/dL. Biopsy was positive for ACTH immunostaining. Patient's condition was deemed to be secondary to neuroendocrine hepatic tumors with bony metastasis to the occipital bone. Diagnosis of Cushing's syndrome secondary to the malignant process explained the patient's persistent anasarca, hyperglycemia, and electrolyte abnormalities.

Nephrology held metolazone and Lasix and started acetazolamide with hydralazine alternatively for BP control given hypokalemia. Metyrapone was initiated after discussing with the endocrinologist. Unfortunately, the patient's strength continued to deteriorate despite working with physical and occupational therapy, requiring max assistance with transfer and inability to stand without assistance. Oncologist was consulted, and they recommended repeat CT head/abdomen/pelvis with and without contrast. CT scan redemonstrated a left occipital lesion, normal size pituitary gland, liver, and adrenal lesions with an interval increase in bilateral pleural effusions, intra- and retroperitoneal edema, and subcutaneous edema consistent with anasarca. Patient developed worsening hypertension, and ECG demonstrated new-onset atrial fibrillation (rate: 80-100 BPM), so metoprolol was initiated for control.

In spite of these interventions, weakness worsened, and the patient progressively became more lethargic and less interactive, eventually refusing participation in therapy, only intermittently spontaneously opening eyes to voice. Modafinil was started for lethargy without success. Palliative care was consulted, and the patient's family opted for comfort care measures with hospice on discharge. Patient passed a week after discharge to hospice.

## 3. Discussion

Hypercortisolism (Cushing's syndrome) is a disorder defined by the excess of glucocorticoids in an individual. The nonspecific symptoms of elevated glucose, obesity, irritability, hypertension, fatigue, muscle wasting, and irregular menstruation are common in the general population [4]. The syndrome most commonly occurs with excess exogenous cortisol administration resulting in adrenal cortical atrophy (not seen in our case). Endogenous causes are subdivided into ACTH-dependent causes, with ACTH greater than 15 pG/mL, and ACTH-independent causes with ACTH less than 5 pg/mL due to feedback inhibition of the pituitary. 60-70% of endogenous hypercortisolism cases are the result of pituitary adenomas, and 5-10% are ectopic sources of ACTH. They can be differentiated by inferior petrosal sinus sampling [5]. Nonneoplastic hypercortisolism, which can occur in depression, anxiety, or alcoholism, must also be considered in ACTH-dependent hypercortisolism [6]. Ectopic ACTH production usually involves small cell carcinoma of the lung, although rarely other sources can be involved.

In our case, we implicated the liver as the source of ectopic ACTH production.  Table 1 demonstrates similar cases previously documented in the literature.

Although our patient had risk factors for nonneoplastic hypercortisolemia with depression and profound stress from learning about metastatic liver lesions, her markedly elevated ACTH 19 times the upper limit of normal gave credence for a nonphysiologic and extensive ectopic source of ACTH production. MRI and multiple CT scans of the brain showed normal pituitary gland size. Although Cushing disease cannot be completely excluded, as inferior petrosal sampling and dynamic testing of corticotropin-releasing hormone stimulation were not completed given the patient's frailty and hospice status, the swift time course of the patient's deterioration was more characteristic of ectopic ACTH neuroendocrine neoplasm. Bilateral nodular hypertrophy of the adrenal glands suggested to primary adrenocortical neoplasm, although primary bilateral macronodular adrenal hyperplasia could not be excluded [12]. Metastatic lesions from the kidney to the liver were unlikely, as the liver is the most common site of metastasis from primary tumors in the colon, lung, and breast. A case could be made that a mixed neuroendocrine nonneuroendocrine carcinoma of the rectum could also present with similar findings; however, imaging suggested against. The more likely pathogenesis of the patient's condition was metastatic lesions to the liver from her former squamous cell carcinoma of the rectum. The metastatic lesion likely underwent metaplasia resulting in neuroendocrine carcinoma that then further metastasized to her bones. Liver lesions resulted in findings of liver failure, with ACTH production and hypercortisolism from neuroendocrine carcinoma contributing to gastric ulcer formation, hyperglycemia, weakness, muscle wasting, anasarca, and depression that accelerated the patient's decline and led to her ultimate demise.

## Figures and Tables

**Table 1:** Literature review of hepatic neuroendocrine carcinoma with Cushing's syndrome.

Primary author	Year	Age (years)	Sex	Primary vs. metastatic
Shah [7]	2007	65	Male	Primary
El Zein [8]	2014	9	Male	Primary
Mineur [9]	2022	50	Male	Metastatic
Waghela [10]	2022	63	Female	Primary
Cipriani [11]	2022	74	Female	Metastatic

## Data Availability

Data supporting this study is not available for readers to access. This is due to legal and ethical concerns regarding patient privacy as abiding by HIPAA. Full information regarding resources is listed for readers to peruse.
